# XGBoost Improves Classification of MGMT Promoter Methylation Status in IDH1 Wildtype Glioblastoma

**DOI:** 10.3390/jpm10030128

**Published:** 2020-09-15

**Authors:** Nguyen Quoc Khanh Le, Duyen Thi Do, Fang-Ying Chiu, Edward Kien Yee Yapp, Hui-Yuan Yeh, Cheng-Yu Chen

**Affiliations:** 1 Professional Master Program in Artificial Intelligence in Medicine, College of Medicine, Taipei Medical University, Taipei City 106, Taiwan; 2 Research Center for Artificial Intelligence in Medicine, Taipei Medical University, Taipei City 106, Taiwan; fychiu7@gmail.com; 3 Faculty of Applied Sciences, Ton Duc Thang University, Ho Chi Minh City 70000, Vietnam; dothiduyen@tdtu.edu.vn; 4 Singapore Institute of Manufacturing Technology, 2 Fusionopolis Way, #08-04, Innovis, Singapore 138634, Singapore; edwardyapp01@gmail.com; 5 Medical Humanities Research Cluster, School of Humanities, Nanyang Technological University, 48 Nanyang Ave, Singapore 639798, Singapore; hyyeh@ntu.edu.sg; 6 Department of Radiology, School of Medicine, College of Medicine, Taipei Medical University, Taipei 11031, Taiwan; 7 Department of Medical Imaging, Taipei Medical University Hospital, Taipei 11031, Taiwan

**Keywords:** radiogenomics, glioblastoma, IDH1 wildtype, O6-methylguanine-DNA methyltransferase, XGBoost, machine learning, F-score feature selection, molecular subtype, concomitant adjuvant temozolomide, noninvasive imaging biomarker

## Abstract

Approximately 96% of patients with glioblastomas (GBM) have IDH1 wildtype GBMs, characterized by extremely poor prognosis, partly due to resistance to standard temozolomide treatment. O6-Methylguanine-DNA methyltransferase (MGMT) promoter methylation status is a crucial prognostic biomarker for alkylating chemotherapy resistance in patients with GBM. However, MGMT methylation status identification methods, where the tumor tissue is often undersampled, are time consuming and expensive. Currently, presurgical noninvasive imaging methods are used to identify biomarkers to predict MGMT methylation status. We evaluated a novel radiomics-based eXtreme Gradient Boosting (XGBoost) model to identify MGMT promoter methylation status in patients with IDH1 wildtype GBM. This retrospective study enrolled 53 patients with pathologically proven GBM and tested MGMT methylation and IDH1 status. Radiomics features were extracted from multimodality MRI and tested by F-score analysis to identify important features to improve our model. We identified nine radiomics features that reached an area under the curve of 0.896, which outperformed other classifiers reported previously. These features could be important biomarkers for identifying MGMT methylation status in IDH1 wildtype GBM. The combination of radiomics feature extraction and F-core feature selection significantly improved the performance of the XGBoost model, which may have implications for patient stratification and therapeutic strategy in GBM.

## 1. Introduction

Glioblastomas (GBMs), the most aggressive and exceptionally invasive brain tumors, are characterized by their frequent resistance to chemotherapy and always recurrence following surgical treatment [1]. Despite extensive efforts worldwide, GBM treatment is considered most challenging in clinical oncology. Approximately 96% of patients with GBM have IDH1 wildtype mutations, and the treatment success rate for these patients (i.e., concomitant adjuvant temozolomide (TMZ) therapy) can be predicted via the O6-methylguanine-DNA methyltransferase (MGMT) gene promoter. MGMT is a DNA repair enzyme that removes the O6 methyl group from methylguanine at the DNA level and thereby it protects alkylating therapeutic agents [2]. It is well known that GBM with MGMT promotor methylation responds to temozolomide better than the unmethylated counterpart. Because GBM with MGMT promotor methylation responds to temozolomide better than the unmethylated counterpart, MGMT methylation status is considered a critical factor of temozolomide resistance and poor progression-free survival. Therefore, a noninvasive imaging biomarker for determining the MGMT promoter status in IDH1-wildtype GBM could lead to improved GBM treatment, with accurate treatment guidance.

Radiomics, a newly emerging method, has been widely adopted to investigate the correlation between clinical symptoms and the underpinning genetic characteristics [3]. Radiomics can quantify tumor phenotypes by extracting extensive features from high-throughput radiographic medical images using data-characterization breakthroughs [4]. Over the past few years, many radiomics models have been developed for different purposes, including predicting the survival rate [5] and distant metastasis [6], and classifying molecular characteristics [7]. Li et al. [8] predicted MGMT promoter methylation status by extracting 1705 multiregional radiomics features from multiparametric MRI; however, this model had a relatively poor predictive ability. Similarly, in an attempt to exploit the full potential of medical imaging, Xi et al. [9] also developed a predictive model in which different sets of radiomics features were investigated. Wei et al. [10] proposed the fusion radiomics signature that can identify MGMT methylation status in patients with World Health Organization grade II–IV astrocytoma, providing an effective preoperative diagnosis for individualized treatment.

One of the most challenging and unanswered issues is to determine an efficient set of radiomics biomarkers that could aid in accurately and promptly predicting MGMT methylation status. Several recent studies have found different radiomics biomarkers such as a combination of 36 features [9], MRI texture features [11], mean, variance, 50th percentile, 90th percentile, Width10–90 APTw values [12], and 6 features [8]. This problem could be resolved via different feature selection techniques such as Boruta algorithm [8], least absolute shrinkage and selection operator (LASSO) regularization [9], or Wilcoxon rank-sum test and multivariate linear logistic regression [13]. Different biomarker sets that have been proposed have demonstrated promising results for the diagnosis and prediction of MGMT methylation status in GBMs. However, their performance is still unsatisfactory, and different radiomics features that can improve the performance as well as provide more information for IDH1 GBM diagnosis and treatment remain unclear. Therefore, we propose a novel radiomics set by using F-score feature selection. Moreover, because IDH1 mutation is related to drug response of GBM [14,15], here, we compare the MGMT status in IDH1 wildtype mutation by using a highly efficient radiomics feature set and advanced machine learning techniques.

In this study, thus, we searched for a model superior to those reported previously by determining the feasibility of a radiomics-based eXtreme Gradient Boosting (XGBoost) model to identify MGMT promoter methylation status in patients with IDH1 wildtype GBM. XGBoost is a widely used and popular tool among different competitions and challenges worldwide because of its potential in controlling overfitting. Handcrafted features were extracted from multimodality MRI, and a set of useful features was used to feed our predictive model. To the best of our knowledge, few studies have experimentally confirmed the effectiveness of XGBoost for this purpose, and thus far, our model is one of the very few models that showed a relatively high performance.

## 2. Results

### 2.1. Patient Characteristics

Table 1 presents the basic characteristics of the study patients. Methylation and unmethylation of the MGMT gene was observed in 26 (49.05%) and 27 (50.94%) patients, respectively. Most patients in our cohort had IDH1 wildtype status. A higher incidence of IDH1 mutation was observed in patients with MGMT methylation. We excluded four patients with IDH1 mutation from the methylated group to treat our analysis as a comparison between MGMT methylation and unmethylation in patients with IDH1 wildtype mutations. Female patients had a higher number of tumors with MGMT promoter methylation (69.23%), whereas male patients had a higher number of tumors without MGMT methylation (70.37%). Four transcriptome subtypes were observed in both groups, with the neural subtype observed in the fewest patients. Moreover, five methylation classes appeared in both groups, and the number of patients in the groups with and without MGMT promoter methylation varied from 1 to 9.

### 2.2. Feature Selection and Radiomics Signature Building

**Figure 1:**
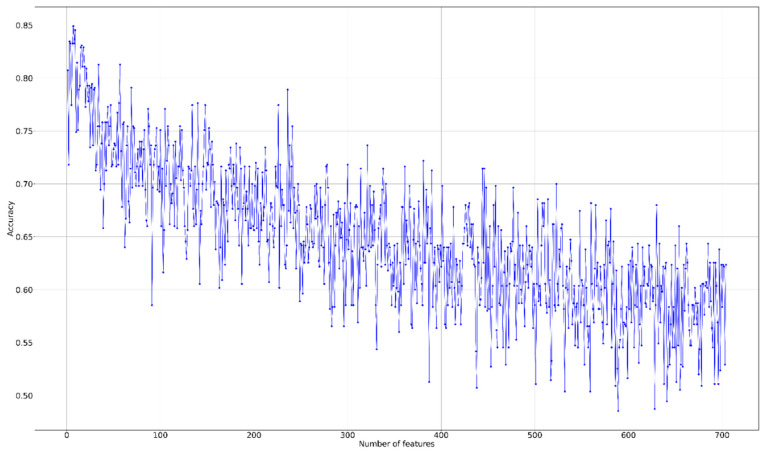
Recursive feature elimination (RFE) curve for predicting O6-Methylguanine-DNA methyltransferase (MGMT) methylation status in patients with IDH1 wildtype glioblastomas (GBM) by using different features. Nine features were used as optimal cutoff points.

We performed an F-score analysis in all features to determine those that might be important for our model. Thereafter, we identified some top-rank features with high F-scores, such as HISTO_ET_T2_Bin6 (F-score = 0.259), TEXTURE_GLRLM_ED_T2_GLV (F-score = 0.1997), and TEXTURE_GLSZM_NET_FLAIR_ZP (F-score = 0.1832). Our subsequent recursive feature elimination (RFE) curve (Figure 1) demonstrated that the first seven, eight, or nine features as input into the model to achieve the optimal results. Here, we used nine features as our cutoff points, and these features are listed in Table 2. These features are our signature to help classify MGMT mutation status in patients with IDH1 wildtype GBM with high accuracy and using as few features as possible.

### 2.3. Supervised Learning Classification

Our model implementation is a binary classification between MGMT methylated and unmethylated status in patients with IDH1 wildtype GBM. After the feature selection step, we inserted the nine selected features into our supervised learning algorithms to observe the differences between their performances. First, to ensure a fair and unbiased comparison between the different methods, tuning the optimal parameters for all the methods is crucial. To address this, we applied the grid search cross validation strategy on the aforementioned algorithms. This is an algorithm to search all the hyperparameter combinations in each model and return the optimal set of combinations. The hyperparameter search range and the optimal sets are presented in Table 3.

**Figure 2:**
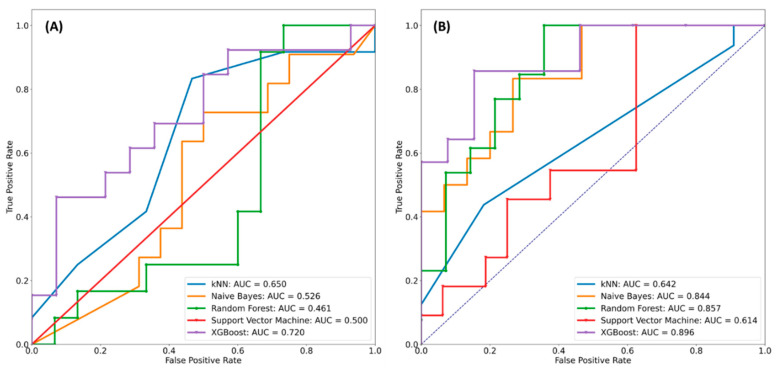
Performance results of different machine learning classifiers in predicting MGMT methylation status. (**A**) All 704 radiomics features. (**B**) Nine top-rank radiomics features.

Our radiomics-based classifiers yielded average accuracies of 58.49%, 69.81%, 79.25%, 67.92%, and 83.02% for k-nearest neighbors (kNN), Naïve Bayes, Random Forest, support vector machine (SVM), and XGBoost, respectively. Thus, XGBoost demonstrated a better performance than the other classifiers. Next, to evaluate the performance at different threshold levels, the receiver operating characteristic (ROC) curves and areas under the ROC curves (AUCs) are required. Figure 2 provides the ROC curves of our model using the aforementioned machine learning algorithms. We observed that the XGBoost classifier outperformed the others on the same level of comparison (AUC = 0.896). It could accurately predict the MGMT status of patients with IDH1 GBM and did not contain many misclassified samples. A comparison of the performance results of all radiomics features showed that the nine top-rank features performed much better than the other features. Therefore, we could use these nine features as our optimal cutoff level for feature selection.

### 2.4. Comparison with Previous Radiomics Studies in Terms of Prediction of O6-Methylguanine-DNA Methyltransferase (MGMT) Status

As different radiomics studies used different data cohorts to validate the performance, fair and accurate comparisons among the different studies can be challenging. However, to evaluate the efficiency of our method, we compared our performance with those of previous works with regard to the prediction of MGMT status. Some related works have yielded promising results in terms of the prediction of MGMT status with different feature selection techniques and radiomics biomarkers. They could be from LASSO [9,16], multivariate linear logistic regression and Wilcoxon rank-sum test [13], or two-tailed Mann–Whitney *U* test [17]. The previous performance results have been retrieved to support the comparison. Table 4 shows the comparative performances between our model and the other state-of-the-art models [9,11,16,17,18,19,20]. According to this comparison, our model had higher sensitivity and specificity than the works of Jiang et al. [16], Crisi et al. [17], Ahn et al. [19], Korfiatis et al. [11], and Sasaki et al. [20]. Moreover, we have also used fewer features (nine features) than the work of Xi et al. [9] (64 features) and reached a better performance. A comparative performance could be seen between our work and Levner et al. [18] while our model reached higher sensitivity and the previous work reached higher specificity. However, our model could reach a little bit better on overall accuracy; and more specifically, our performance results were more balance between sensitivity and specificity. Therefore, the aforementioned evidence shows that our model was observed to be superior to the other models at the same level of comparison.

## 3. Discussion

Recently, as the availability of public medical imaging datasets (e.g., TCGA and TCIA) by which patient information is extracted from different angles increases [21], it becomes possible to considerably improve the predictive efficiency of molecular subtype, in general, and MGMT genotype, in particular, by fusing information originating from the correlation of high-throughput data with radiomics (or transcriptomics, proteomics, etc.). Through studies on the dataset of 53 patients with GBM, we retrospectively investigated a new radiomics model for predicting the MGMT methylation status in patients with IDH1 wildtype GBM. The model uses nine readily available variables and has high predictive accuracy with XGBoost classifier. Our internal sampling showed the strong predictive ability of the model. Moreover, the high AUC values (Figure 2) indicate that this predictive model can be widely and accurately used for the evaluation of the therapeutic effect of MGMT methylation in IDH1 wildtype GBM. It also demonstrates the significant differences between IDH1 wildtype MGMT methylation and unmethylation groups in terms of their radiomics features.

Conventionally, different feature selection methods in the radiomics domain have been extensively used to predict MGMT methylation status, such as LASSO [9,16], two-tailed Mann–Whitney *U* test [17], chi-square test [19], or principal component analysis (PCA) [20]. However, we evaluated the ability of F-score feature selection in finding an optimal set of radiomics features. We observed that the important radiomics features could be explained from the original dataset with their means and variances, but not from the other unsupervised learning techniques. Thus, our radiomics features are reliable and more suitable to our selected model. To the best of our knowledge, this is the first study in which F-score feature selection has been applied to evaluate MGMT methylation status in particular and GBM in general. Furthermore, we validated different machine learning techniques to determine the models that provided information from the optimal radiomics features set. The current results indicated that XGBoost is superior to other methods for predicting MGMT genotype in patients with IDH1 wildtype glioblastomas.

Our F-score analysis and XGBoost algorithm established a novel set of nine radiomics features for prediction of IDH1-wildtype MGMT status. Our radiomics features differ from those observed in the previous works on GBM using traditional feature selection techniques. We even used less number of radiomics features than the previous works [9,16,17] did. The optimal set containing nine radiomics features (Table 3) might attract much attention for further research on the prediction of MGMT methylation status of IDH1 GBMs. These different radiomics features generating from our study could become a potential biomarker for predicting MGMT methylation status accurately. Seven of the nine features in our biomarker set were from textural analysis. It has been shown that textural features were associated with MGMT methylation status in particular and might be extended for different molecular subtypes in glioma. The efficiency of textural features has also been proven in radiomics-based GBM problems on MRI such as prediction of 1p/19q-codeletion status [22], IDH1 mutation [23], or even MGMT methylation status [11,18]. Furthermore, the wavelet transform (i.e., GLSZM) appeared to be mostly in the important feature set. In some cases, enhancement or edema was not observed on MRI results of some patients with diffuse glioma. Therefore, noninvasive and preoperative prediction of the MGMT genotype in patients with IDH1-wildtype GBM by using computational model is possible with the help of wavelet transform features or nonenhanced part of the tumor core (NET)-hit features.

Despite the promising results of this study, it also has certain limitations. First, in order to improve the quality of this research, a larger sample size and external validation cohort are important to assess the generalizability of our model. However, this study addressed this limitation to a certain extent by repeating the training several times via leave-one-out cross-validation (LOOCV). In addition, hyperparameter optimization is an important step in machine learning to help the model reach its optimal result as well as to avoid overfitting. As shown in Table 3, we searched several parameter sets, and each model achieved its optimal results. Second, some recent studies have shown that deep learning has the potential to generate deep radiomics features in a noninvasive manner and to identify molecular genotype for gliomas [24,25]. It seems possible to improve the classification performance of our model by implementing more imaging features that originated from these deep neural networks. Another promising solution is the integration of IDH mutation status in the prediction of MGMT status. As shown previously [26], MGMT promoter methylation status can still be identified using a sophisticated method in a group of patients with IDH wildtype mutations. Therefore, additional studies could incorporate radiomics and IDH wildtype status to boost the predictive performance of this model. Furthermore, transfer learning from single-modality datasets could be considered promising for research that is more comprehensive in the future because it may be helpful for training the multimodal fusion networks. Finally, it is also imperative to extend the number of paired datasets for more rigorous analyses and comparisons.

## 4. Materials and Methods

### 4.1. Patient Cohort

The datasets considered for analyzing the methods have been retrieved from The Cancer Imaging Archive (TCIA) [21], a comprehensive resource for cancer imaging data. Since all the datasets were public data and have been used in a variety of published works, the Institutional Review Board for ethical issues in research approved them. Various cancer projects are deposited in TCIA, and this study has selected patients with GBM from the TCGA-GBM project, which included preoperative multimodal MRI (mMRI) scans from 262 participants. The entire radiological dataset of the TCGA-GBM cohort comprises 262 mMRI scans collected from eight institutions: Henry Ford Hospital (Detroit, MI, USA), CWRU School of Medicine (Cleveland, OH, USA), University of California (San Francisco, CA, USA), Emory University (Atlanta, GA, USA), MD Anderson Cancer Center (Houston, TX, USA), Duke University School of Medicine (Durham, NC, USA), Thomas Jefferson University (Philadelphia, PA, USA), and Fondazione IRCCS Instituto Neuroligico C. Besta (Milan, Italy). Thereafter, this study included only the subset of the preoperative standard scans of this cohort, in which the available MRI modalities of at least precontrast T1-weighted (T1) image, gadolinium-enhanced T1-weighted (T1-Gd) image, T2-weighted image, and T2-FLAIR image were selected.

MGMT DNA methylation status was clinically determined on the basis of CpG islands, as described in previous studies [27,28]. In brief, frozen tissue samples were used to isolate DNA in which 1.0 µg of the total DNA sample was transformed by bisulfite using the EZ DNA Methylation Kit (#D5001; Zymo Research Corporation, Irvine, CA, USA). Next, the HM-450K (Illumina—San Diego, CA, USA) was used to analyze the DNA methylation status, as suggested by the Genomics platform at the University of Geneva. One of each of the examined CpG two site-specific probes is specifically created for the methylated locus and the other, for the unmethylated locus, where the chemically transformed DNA is hybridized. To allow the assessment of methylated and unmethylated alleles, the single-base enlargement of these hybridized probes is specifically bound to the single-base enlargement of these hybridized probes. According to the analysis conducted in a previous study [27], there were 53 GBM patients that had information of MGMT methylation status as well as the IDH1 status. Therefore, we extracted these patients (26 patients with GBM with methylated MGMT and 27 patients with GBM with unmethylated MGMT) for inclusion in our study cohort.

### 4.2. MRI Segmentation and Radiomics Features

**Figure 3:**
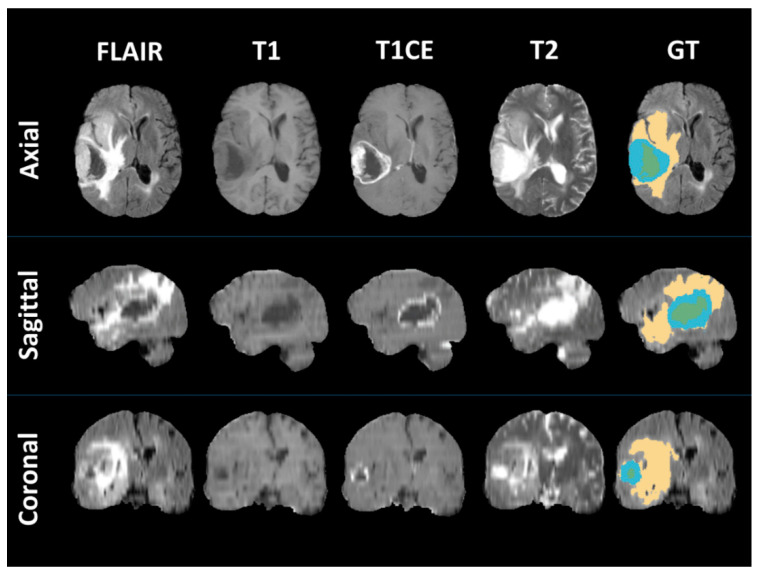
An illustrative example of how to segment the MRI scan of a patient with GBM into three different regions (non-enhanced part of tumor core (NET): green, enhanced part of tumor core (ET): blue, peritumoral edema (ED): yellow, GT: ground truth).

The MRI scans were segmented according to the criteria of the Brain Tumor Segmentation (BraTS) challenge [29]. According to this criterion, brain tumors could be classified into three sub-regions: (1) the enhanced part of the tumor core (ET) is characterized on the basis of areas displaying hyperintensity in T1-Gd, when compared with both T1 and normal/healthy white matter (WM) in T1-Gd. Areas showing contrast leakage of disrupted blood–brain barriers (often observed in high-grade gliomas) are represented using ET; (2) the nonenhanced part of the tumor core (NET) is used to represent regions that show no enhancement and particularly nonenhanced transitional/prenecrotic and necrotic regions in the tumor core that are usually resected in addition to the ET. The NET shows hypointense appearance in T1-Gd in comparison with both T1 and normal/healthy WM in T1-Gd; and (3) finally, a hyperintense signal on the T2-FLAIR volumes is used to describe the peritumoral edema. In this study, we used the benchmark segmentations from a previous study [30], whereby the authors proposed standard segmentations (manually by radiologist experts) as well as radiomics features for TCGA-GBM studies. Figure 3 shows an example of how the ground truth of GBMs has been segmented.

Our model was trained with the set of features that were volumetrically extracted and included (i) intensity information, (ii) image derivative, (iii) geodesic information, (iv) texture features, and (v) GLISTR posterior probability maps [31]. A total of 704 radiomics features were extracted. We evaluated the performance among all features, and the radiomic features with high performance were used in our final model.

### 4.3. Radiomics Feature Selection

The most crucial concern in machine learning-based radiomics is the high dimension of data. The many radiomics features fed into machine learning models increase the complexity of the models and could lead to overfitting issues as well. Therefore, reducing the number of features is essential, which can be accomplished using various existing methods, such as PCA and clustering (K-means or hierarchical). However, in this study, we tested the possibility of F-score feature selection in identifying the optimal features.

To evaluate binary classifications and their confusion matrices, F-score is a simple measure of the accuracy of a test, in which two sets of real numbers are involved [32]. Given training vectors *x_k_*, and *k* = 1,…,m, if the number of positive (methylated MGMT status) and negative instances (unmethylated MGMT status) are *n*_+_ and *n*_−_, respectively, the F-score is described in the following equation:

(1) $$F\left(i\right)=\frac{{\left({\bar{x}}_{i}^{\left(+\right)}-{\bar{x}}_{i}\right)}^{2}+{\left({\bar{x}}_{i}^{\left(-\right)}-{\bar{x}}_{i}\right)}^{2}}{\frac{1}{n_{+}-1}\sum_{k=1}^{n+}{\left(x_{k,i}^{\left(+\right)}-{\bar{x}}_{i}^{\left(+\right)}\right)}^{2}+\frac{1}{n_{-}-1}\sum_{k=1}^{n-}{\left(x_{k,i}^{\left(-\right)}-{\bar{x}}_{i}^{\left(-\right)}\right)}^{2}},$$

where ${\bar{x}}_{i}$, ${\bar{x}}_{i}^{\left(+\right)}$, and ${\bar{x}}_{i}^{\left(-\right)}$ are the averages of the *i*^th^ feature of the whole, positive, and negative datasets, respectively, and $x_{k,i}^{\left(+\right)}$ and $x_{k,i}^{\left(-\right)}$ are the *i*^th^ feature of the *k*^th^ positive and negative instance, respectively. F-score values of 704 radiomics features were first computed and then ranked from the largest to smallest to observe the top-important features. Next, RFE was performed to fit our model and remove the weakest feature (or features) until the specified number of features was reached. Thereafter, the threshold point, which yielded the best performance, was selected as our optimal cutoff point for feature selection.

### 4.4. Machine Learning

Machine learning is a popular technique in the field of artificial intelligence, which enables automatic extraction of salient image features from extensive datasets, leading to a more precise prediction [33]. Many studies have demonstrated that machine learning achieves a promising performance in tumor segmentation, tumor classification, and survival or genotype prediction [34,35,36,37]. In the radiomics field, machine learning algorithms (both supervised and unsupervised learning) could help to determine the informative radiomics features, thereby accurately predicting the outcomes. We attempted five classification algorithms in this study, and on the basis of their performance, we subsequently selected the best algorithm to build the final classification model. Among the five different classifiers, kNNs and Naïve Bayes are simple algorithms on the basis of distance function learning and Bayes theorem, respectively. Next, SVM is a common machine learning algorithm based on the notion of decision plane, and it is primarily used for the classification tasks. Its goal is to determine the optimal dividing hyperplane that maximizes the margin of the training data. The last algorithms are both ensemble methods including Random Forest and XGBoost that ensemble the individual outcome predictions, and the model that receives the most votes becomes the final model. We also performed a grid search on cross-validation to determine the best optimal parameters for all aforementioned machine learning algorithms.

### 4.5. Statistical Analysis

Owing to our limited data, LOOCV has been used to legitimize the overall performance. That is, each sample will be used as the testing set, and the others as the training set. The process will be looped again, in which every sample in our dataset becomes testing set once. The final reported accuracy is the mean of all testing accuracy values. Because our problem is a binary classification, the performances of the predictive models were firstly measured by sensitivity, specificity, and accuracy. These data are presented as the percentage of the correct predictions on a different set of data such as positive, negative, and all data. These evaluation measurements are defined in previous works on different aspects of biomedical tasks [38,39] as follows:

(2) $$\mathrm{Sensitivity}=\frac{\mathrm{TP}}{\mathrm{TP}+\mathrm{FN}},$$

(3) $$\mathrm{Specificity}=\frac{\mathrm{TN}}{\mathrm{TN}+\mathrm{FP}},$$

(4) $$\mathrm{Accuracy}=\frac{\mathrm{TP}+\mathrm{TN}}{\mathrm{TP}+\mathrm{FP}+\mathrm{TN}+\mathrm{FN}},$$

where TP, TN, FP, and FN denote true positives, true negatives, false positives, and false negatives, respectively. Moreover, to overcome the possibilities of the imbalance dataset, we reported the ROC curve and AUC values to observe the overall performance at different threshold points.

## 5. Conclusions

Through this study, we investigated the role of MRI features and radiomics-based XGBoost model in predicting MGMT genotype in patients with IDH1 wildtype GBM. F-score has been used as an efficient technique to select the optimal radiomics features for prediction of MGMT methylation status. Thereafter, XGBoost, with the top nine ranking features, achieved the highest predictive performance and stability (accuracy, 0.887 and AUC, 0.896). Our radiogenomics model could maximize the value of the information contained in the medical images. The recognition of the optimal radiomics-based machine learning model for noninvasive and preoperative prediction of MGMT promotor status may be beneficial in the primary diagnosis and treatment plan for patients with IDH1 wildtype GBMs. The study suggests that the combination of F-score feature selection and XGBoost is a promising fit for prediction of IDH1-wildtype MGMT status, in particular, as well as radiomics problems, in general. Additional radiomics studies could exploit this combination to improve the predictive performance of the model.

## Figures and Tables

**Table 1:** Patient characteristics (*n* = 53).

	Methylated (*n* = 26)	Unmethylated (*n* = 27)
Age (mean ± SD, years)	53 ± 16.43	60.83 ± 12.12
Gender		
Male	8	19
Female	18	8
TCGA subtype		
Classical	6	8
Mesenchymal	9	8
Neural	1	2
Proneural	6	9
Methylation class		
CL_1	1	4
CL_2	8	9
CL_3	6	5
CL_4	4	5
CL_6	3	4

**Table 2:** Top nine radiomics features in our model.

No.	Feature Name	Modality	Matrix	Type
1	HISTO_ET_T2_Bin6	T2	First Order	Histogram
2	TEXTURE_GLRLM_ED_T2_GLV	T2	GLRLM	Texture
3	TEXTURE_GLSZM_NET_FLAIR_ZP	FLAIR	GLSZM	Wavelet Texture
4	TEXTURE_GLSZM_NET_FLAIR_SZE	FLAIR	GLSZM	Wavelet Texture
5	TEXTURE_GLSZM_NET_FLAIR_ZSN	FLAIR	GLSZM	Wavelet Texture
6	TEXTURE_GLSZM_NET_T1_ZSN	T1	GLSZM	Wavelet Texture
7	TEXTURE_GLSZM_NET_T1_SZE	T1	GLSZM	Wavelet Texture
8	HISTO_ED_T2_Bin5	T2	First Order	Histogram
9	TEXTURE_GLSZM_NET_T1_ZP	T1	GLSZM	Wavelet Texture

GLRLM: Gray Level Run Length Matrix, GLSZM: Gray Level Size Zone Matrix.

**Table 3:** Hyperparameter search range and optimal values for each learning algorithm.

Learning Algorithm	Hyperparameter Range	Optimal Value
K-nearest neighbors (kNN)	n_neighbors = [1, 2, 3, .., 30]	1
	weights = [uniform, distance]	uniform
	metric = [euclidean, manhattan, minkowski]	minkowski
Random Forest	max_depth = [10, 20, 30, 40, 50, …, 100, 110, None]	None
	max_features = [‘auto’, ‘sqrt’]	sqrt
	min_samples_leaf = [1, 2, 3, 4, 5]	1
	min_samples_split = [2, 4, 6, 8, 10, 12]	10
	n_estimators = [100, 200, 300, 400, 500, 600, …, 2000]	2000
Support vector machine (SVM)	C = [0.001, 0.01, 0.1, 1, 10]	0.001
	gamma = [0.001, 0.01, 0.1, 1]	0.1
	kernel = [rbf, linear, poly, sigmoid]	poly
eXtreme Gradient Boosting (XGBoost)	min_child_weight = [1, 2, 3, 4, 5, 6, 7, 8, 9, 10]	1
	gamma = [0.5, 1, 1.5, 2, 2.5, 3, 3.5, 4, 4.5, 5]	2.5
	subsample = [0, 0.2, 0.4, 0.6, 0.8, 1]	0.6
	colsample_bytree = [0, 0.2, 0.4, 0.6, 0.8, 1]	0.4
	max_depth = [10, 20, 30, 40, 50, …, 100, 110, None]	50
	n_estimators = [100, 200, 300, 400, 500, 600, …, 2000]	200

**Table 4:** Comparison between our models and previously published works in predicting MGMT mutation status in GBMs.

	Biomarkers	Classifiers	Sensitivity	Specificity	Accuracy
Jiang et al.	15 features	Mann–Whitney test	0.821	0.857	0.886
Levner et al.	8 features	L1-regularized neural networks	0.854	0.9	0.877
Xi et al.	63 features	Support vector machine	0.888	0.838	0.866
Crisi et al.	14 features	Multilayer perceptron	0.75	0.85	-
Ahn et al.	-	Mann–Whitney *U*-test	0.563	0.852	-
Korfiatis et al.	4 features	Support vector machine	0.803	0.813	-
Sasaki et al.	5 features	LASSO	0.67	0.66	0.67
Our study	9 features	XGBoost	0.88	0.887	0.887

LASSO: least absolute shrinkage and selection operator, “-” indicates that this value has not been reported in the original paper.
